# Evaluation of uNGAL and TIMP-2*IGFBP7 as early biomarkers of Acute Kidney Injury in Caucasian term and preterm neonates: a prospective observational cohort study

**DOI:** 10.1186/s13052-025-01899-8

**Published:** 2025-03-01

**Authors:** Raffaella Panza, Annalisa Schirinzi, Maria Elisabetta Baldassarre, Rossella Caravita, Riccardo Laterza, Elisa Mascolo, Federica Malerba, Francesca Di Serio, Nicola Laforgia

**Affiliations:** 1 Neonatology and Neonatal Intensive Care Unit, Department of Interdisciplinary Medicine, “Aldo Moro” University of Bari, Bari, Italy; 2 Clinic Pathology Unit, University Hospital Policlinico, Bari, Italy

**Keywords:** Acute kidney injury [Mesh], Biomarkers [Mesh], Neonates [Mesh], NGAL, TIMP-2*IGFBP7, Preterm birth [Mesh]

## Abstract

**Background:**

Early diagnosis of Acute Kidney Injury (AKI) in neonates is a complex challenge. Novel urinary biomarkers such as uNGAL and TIMP-2*IGFBP7 may be helpful for predicting AKI earlier than changes in serum creatinine (sCr) and urinary output (UOP) in the neonatal period. uNGAL is a marker of tubular injury and its concentration rises immediately after AKI, while the proteins TIMP-2 and IGFBP7 jointly participate in the G1 phase cell cycle arrest processes and their tubular expression and urinary excretion increase in response to kidney damage. The aim of this study is to determine urinary concentrations of uNGAL and TIMP-2*IGFBP7 in term and preterm newborns and to evaluate their predictive role of AKI.

**Methods:**

Forty-two heathy term neonates and twenty-six preterm infants were prospectively recruited at the NICU of Policlinico in Bari, Italy. uNGAL and TIMP-2*IGFBP7 were measured in fresh urinary samples collected via perineal bag either before discharge (term neonates) or over the first week of life (preterm neonates).

**Results:**

In term neonates median uNGAL and TIMP-2*IGFBP7 concentrations were 41.40 ng/ml (IQR 20.25–74.5) e 0.22 (ng/ml)^2^/1000 (IQR 0.14–0.34), respectively. In preterm infants without AKI, uNGAL median concentrations over the first week of life ranged between 10 and 16 ng/ml, whereas median concentration of TIMP-2*IGFBP7 ranged between 0.05 and 0.08 (ng/ml)^2^/1000. Preterm infants who developed AKI during the first week of life had significantly higher uNGAL median concentrations compared to preterm infants without AKI (148.5 vs. 10.0, *p* = 0.04; 324.0 vs. 15.75, *p* = 0.02; 318.0 vs. 16.0 ng/ml, *p* = 0.04). Conversely, TIMP-2*IGFBP7 did not significantly increase in preterm infants with AKI. Preterm female neonates without AKI had significantly higher uNGAL than male neonates (46.5 vs. 10.0 ng/ml; Mann-Whitney U-test, *p* =0.013).

**Conclusions:**

Our data show that uNGAL could be more useful than TIMP-2*IGFBP7 for early detection of AKI in preterm newborns. Further studies are needed to evaluate the role of both biomarkers during AKI and their relationship with gender, gestational age and birth weight.

**Supplementary Information:**

The online version contains supplementary material available at 10.1186/s13052-025-01899-8.

## Implication for practice

Acute Kidney Injury (AKI) in neonates is an underestimated clinical issue, mainly for the lack of a univocal definition among neonatologists. Novel urinary biomarkers, such as uNGAL and TIMP-2*IGFBP7, have been suggested, but their use in neonatal clinical practice is still limited.

We performed a single-center prospective observational cohort study that shows, in healthy term neonates, median concentrations of uNGAL and TIMP-2*IGFBP7 of 41.40 ng/ml (IQR 20.38–75.50) and 0.23 (ng/ml)^2^/1000 (IQR 0.15–0.37), respectively.

In preterm infants without AKI, uNGAL median concentrations, over the first week of life, ranged between 10 and 16 ng/ml, whereas median concentration of TIMP-2*IGFBP7 ranged between 0.05 and 0.08 (ng/ml)^2^/1000.

Preterm infants who developed AKI during the first week of life had significantly higher uNGAL median concentrations, whereas TIMP-2*IGFBP7 did not increase significantly.

On the basis of our findings, uNGAL seems more useful than TIMP-2*IGFBP7 for the diagnosis of neonatal AKI.

Gender affects uNGAL, making it difficult to identify a uniform cut-off for neonatal population.

**Supplementary Information:**

The online version contains supplementary material available at 10.1186/s13052-025-01899-8.

## Introduction

Acute Kidney Injury (AKI) is a complex disease with significant morbidity and mortality both in adult and pediatric patients [1, 2]. According to the Assessment of Worldwide Acute Kidney Injury Epidemiology in Neonates (AWAKEN), AKI occurs in 29.9% of newborns younger than 14 days admitted to neonatal intensive care units (NICUs), especially preterm neonates [3], due to their immature renal structure and function and the frequent use of nephrotoxic drugs [4]. Common causes of neonatal AKI are perinatal asphyxia, congenital anomalies of kidney and urinary tract (CAKUT), congenital heart diseases, sepsis and medical treatment of a hemodynamically significant patent ductus arteriosus (PDA) [5].

Currently, the diagnosis of AKI is based on elevation of serum creatinine (sCr > 1.5 mg/dl) or decrease in urinary output (UOP < 0.5 ml/kg per hour) [6], according to the Kidney Disease Improving Global Outcomes (KDIGO) classification, modified for the neonatal population by Jetton and Askenazi [7]. However, both sCr and UOP are imprecise and untimely markers of renal function in the neonatal age. Serum creatinine is a marker of renal function, not a marker of damage [8] and, after injury, its increase is delayed by 48–72 h, when renal function could be already compromised. Soon after birth, neonatal sCr reflects maternal levels and then it decreases at a pace dependent on gestational age (GA) at birth [9]. Besides, sCr determination requires multiple blood samplings which are undesirable in neonates in order to reduce pain and risk of iatrogenic anaemia.

UOP evaluation has also some limits. AKI in neonates is frequently non-oliguric, so that oliguria (i.e. UOP < 0.5 ml/kg/h) is a low sensitive marker of AKI. Common therapies (e.g. volume expanders, diuretics, vasodilating agents or aggressive fluid restoration) may also misleadingly modify UOP [10]. Moreover, UOP is generally assessed by weighing nappies, a non-invasive but inaccurate method [11], since invasive assessment by urinary catheter is burdened by the risk of nosocomial infections and therefore limited for critical or unstable neonates [12].

New early biomarkers of AKI have recently been suggested, including Lipocalin-2 (LCN2, known as Neutrophil Gelatinase-associated Lipocalin - NGAL) [13], Tissue Inhibitor of Metalloproteinase-2 (TIMP-2), and Insulin-like Growth Factor-Binding Protein 7 (IGFBP7) [14, 15].

NGAL is a 25 kDa protein expressed by numerous cells, including epithelial cells and neutrophils. It is a bacteriostatic agent that interferes with siderophore-mediated iron acquisition [16]. Renal tubular cells produce NGAL early in response to various stressful situations, in order to protect the site from oxidative stress and activate iron-dependent repair/regeneration systems. NGAL has a very short half-life (15–20 min), hence it is associated with tubular damage and not with impaired glomerular filtration [13]. It is detectable in both plasma and urine.

TIMP-2 inhibits the activity of matrix metalloproteinase (MMP), but also interferes with cell cycle regulation. IGFBP7 is a secreting protein of the Insulin-like Growth Factor-Binding Protein (IGFBP) superfamily and regulates the bioavailability of Insulin-like Growth Factors (IGFs) through direct low affinity bonds [17]. Following damage, IGFBP7 and TIMP-2 are expressed in the renal tubular cells, thereby resulting in up-regulation of tumor-suppressor genes and G1 cell cycle arrest for short periods of time [15], in order to prevent mitosis in the presence of damaged DNA [18].

The urinary complex TIMP-2*IGFBP7 proved effective in predicting the development of severe or moderate AKI in high-risk patients with significant accuracy and stability [19], and its clinical applications have rapidly increased [18] since the Food and Drug Administration (FDA) approval of the test “NephroCheck^®^” in patients 21 years of age or older in 2014.

However, the employment of novel urinary biomarkers of AKI is still not widespread in neonatal settings, also because of the lack of neonatal reference limits.

The aim of the present study is to measure, both in term and preterm newborns, urinary NGAL (uNGAL) and TIMP-2*IGFBP7 and to evaluate their modifications in preterm neonates developing AKI during the first week of life.

## Methods

Single-center prospective observational cohort study conducted in two phases at the Neonatal Intensive Care Unit of the Policlinico Hospital in Bari, Italy. The STrengthening the Reporting of OBservational studies in Epidemiology (STROBE) guidelines for reporting of observational studies were followed [20].

- Phase 1–1st to 30th June 2019: full-term newborns;
- Phase 2–1st May to 31st July, 2020 and 1st March to 30th April, 2021: preterm newborns.

The research protocol was approved by the local ethical committee (number 65290—date 30/07/2019).

### Study population

1. Cohort of healthy full-term inborn newborns;
2. Cohort of preterm infants, for whom the following inclusion criteria were applied:

- Gestational age at birth: 26 + 0 to 36 + 6 weeks;
- Inborn neonates and outborn transferred within the first 72 h of life.

The exclusion criteria for both cohorts were:

- urinary (CAKUT) and/or major congenital malformations;
- major comorbidities and/or clinical instability in the first week of life (e.g. asphyxia, early neonatal sepsis);
- inability to collect urine samples;
- outborn neonates transferred after the first 72 h of life;
- length of hospitalization < 8 days of life.

Parental informed consent was obtained for all patients. For each newborn, both maternal and neonatal demographic and anamnestic data regarding pregnancy, delivery and the postnatal period were collected. All datasets were anonymous. Data regarding significant neonatal morbidity were also collected, including the use of nephrotoxic drugs (i.e. aminoglycoside antibiotics such as Amikacin and Gentamicin, glycopeptide antibiotics like Vancomycin and nonsteroidal anti-inflammatory drugs such as Ibuprofen), the duration of invasive and non-invasive ventilatory support and oxygen supplementation, occurrence of major adverse events such as early or late onset sepsis, necrotizing enterocolitis (NEC) Bell’s stage > 2, grade 3–4 intraventricular hemorrhage (IVH), periventricular leukomalacia (PVL), posthemorrhagic hydrocephalus, retinopathy of prematurity (ROP) stage 3–4 or requiring laser therapy, bronchopulmonary dysplasia (BPD), defined as the need for supplementary oxygen therapy at 28 days of life and/or 36 weeks GA, hemodynamically significant PDA, death.

### Primary and secondary outcomes

The primary outcome was to determine values of the urinary biomarkers uNGAL and TIMP-2*IGFBP7 in full-term newborns during the first three days of life (phase 1) and in preterm infants during the first week of life (phase 2).

Preterm neonates were further divided into two groups based on the diagnosis of AKI, according to the modified KDIGO criteria for the neonatal population [7].

The prespecified secondary outcomes were: influence of demographic factors (gender, gestational age and birth weight - BW) in both groups; predictive role of AKI, defined according to the modified KDIGO criteria for the neonatal population [7], of both the urinary biomarkers uNGAL and TIMP-2*IGFBP7, during the first week of life.

### Sample collection and evaluation of renal function

Fresh urinary samples of 3–5 ml were collected non-invasively using Pediatric urine bags (100 ml - RAYS S.p.A. Osimo– Italy) before discharge in full-term infants and at 3 time points (days of life, DOL, 1–3; 4–5; 6–8) in preterm neonates. Urinary samples were then transferred to a clean centrifuge tube without additives. After centrifugation for 10 min at 1000 x g the supernatant was transferred to a clean receptacle. In each preterm newborn, sCr levels from central venous or arterial blood samples during the first week, after the first 24 h of life and daily urinary output were assessed to define the KDIGO stage.

### Assay methods

Measurement of uNGAL was performed on Architect i1000 instrument (Abbott Laboratories, Wiesbaden, Germany). The Architect NGAL assay is a two-step chemiluminescent microparticle immunoassay, which has a limit of detection (LoD) of < 15 ng/ml and an imprecision (CV%) of < 10% as declared by manufacturer. Prior to any measurement of uNGAL, urine samples were centrifuged for 5 min at 190 rpm at room temperature. Measurement of TIMP-2 and IGFBP7 was performed by NephroCheck^®^ Test on the Astute140 Meter (Astute Medical, San Diego, CA, USA). The NephroCheck^®^ Test is a quantitative, fluorescence, lateral flow immunoassay technology. The test contains sandwich immunoassays for TIMP-2 and IGFBP7 in a single-use plastic test cartridge. Urine samples are centrifuged, added to a buffer, and mixed with a fluorescent antibody conjugate prior to measurement. The sample is then applied to the cartridge and inserted into a bench-top instrument that reads the fluorescent signals from each of the TIMP-2 and IGFBP7 immunoassays. The Astute140 Meter converts the fluorescent signals into a single numerical result that is the product of the urinary concentrations of [TIMP-2]*[IGFBP7] and is called AKIRisk^®^ Score ([(TIMP-2]*[IGFBP7])/1000, units = (ng/mL)^2^/1000) [21].

### Statistical analysis

Continuous quantitative variables, for which the normality of distribution was initially verified by the Shapiro-Wilk test, were expressed by indicating the mean and the standard deviation (SD) in the case of Gaussian distributions and by means of median and interquartile range (IQR) in the case of non-normal distributions; categorical variables were expressed in terms of proportions.

To compare the variables uNGAL and TIMP-2*IGFBP7 at the 3 time points, the Friedman test (non-parametric) was used; where significant, Wilcoxon’s signed-rank test for paired samples was applied for each comparison between two time points.

Comparison of the variables uNGAL and TIMP-2*IGFBP7 between groups defined on the basis of dichotomous qualitative variables (gender, AKI, GA < or > 32 weeks, BW < or > 1500 g) was evaluated by Mann-Whitney test for unpaired samples and represented by boxplot.

The presence of an association between the variables uNGAL and TIMP-2*IGFBP7 for each collection time and the other continuous quantitative variables observed in the study was evaluated by means of Spearman correlation analysis.

Multiple comparisons among term, preterm non-AKI and preterm AKI groups on the basis of quantitative demographic variables were evaluated by Independent-Samples Kruskal-Wallis Test. Significance values have been adjusted by the Bonferroni correction for multiple tests.

In preterm infants, the secondary outcomes were compared between the AKI and non-AKI groups by Mann-Whitney test for unpaired samples and Fisher’s exact test, with Bonferroni’s correction on multiple comparisons.

Finally, the performance as predictors of AKI of the two urinary markers, uNGAL and TIMP-2*IGFBP7, was evaluated through ROC curves, identifying the optimal cut-off for each, based on the Youden’s Index. The statistical significance of *p* < 0.05 was accepted for all tests.

Data were collected from medical notes and Neocare software (GPI SpA, Trento, Italy). The completed forms have been inserted into a database created with Office Microsoft^®^ Excel software version 16.53. The statistical analysis of the data was carried out using the IBM SPSS Statistics v.26 software and the R version 4.0.1 software.

## Results

### Enrollment and demographics

Forty-two healthy full-term and twenty-six preterm newborns were enrolled (Fig. S1).

The diagnosis of AKI according to the modified KDIGO criteria for the neonatal period [7] was made in three (11.5%) preterm infants. Neonatal and maternal demographic characteristics of the three groups are shown in Table 1.

**Table 1:** Neonatal and maternal demographics

	Term *n* = 36^#^	Preterm non-AKI *n* = 23	Preterm AKI *n* = 3	Kruskal- Wallis Test Significance
Male sex, n (%)	24 (66.7)	15 (65.2)	2 (66.7)	*p* = 1.0
GA weeks, median (IQR)	39.8 (39.0-40.8)	32.3 (31.0-33.9)	27.4 (26.8–27.5)	*p* < 0.0001*
GA < 32 weeks, n (%)	0 (0)	9 (39.1)	3 (100)	*p* < 0.0001*
AGA, n (%)	27 (75)	22 (95.7)	3 (100)	*p* > 0.05
LGA, n (%)	3 (8.3)	0 (0)	0 (0)	*p* > 0.05
SGA, n (%)	6 (16.7)	1 (4.3)	0 (0)	*p* > 0.05
Birth weight g, median (IQR)	3300.0 (3002.5–3605.0)	1560.0 (1400.0-1922.5)	1035.0 (992.5-1072.5)	*p* < 0.0001*
Birth weight < 1500 g, n (%)	0 (0)	9 (39.1)	3 (100)	*p* < 0.0001*
Weight loss %, median (IQR)	7.2 (5.0-8.8)	6.0 (4.3–9.6)	9.7 (9.6–10.7)	*p* = 0.12
Multiple pregnancy, n (%)	2 (5.6)	12 (52.2)	0 (0)	*p* < 0.0001^§^
Apgar score 1 min, median (IQR)	9.0 (9.0–9.0)	8.0 (7.0–8.0)	5.0 (4.0-6.5)	*p* < 0.0001*
Apgar score 5 min, median (IQR)	10.0 (10.0–10.0)	9.0 (8.0–9.0)	8.0 (8.0-8.5)	*p* < 0.0001*
Maternal age, median (IQR)	35.0 (27.5–38.0)	33.0 (30.0–39.0)	37.0 (33.0–38.0)	*p* = 0.9
White race, n (%)	33 (94.3)	23 (100)	3 (100)	*p* = 0.56
Primiparous, n (%)	10 (30.3)	13 (56.5)	1 (33.3)	*p* = 0.14
Antenatal steroids, n (%)	4 (11.1)	20 (87.0)	3 (100)	*p* < 0.0001*
Caesarean section, n (%)	12 (33.3)	20 (87.0)	2 (66.7)	*p* < 0.0001^**§**^
PROM > 24 h, n (%)	0 (0)	5 (21.7)	1 (33.3)	*p* < 0.0001*
PIH, n (%)	2 (5.6)	2 (8.7)	0 (0)	*p* = 0.71
Pre-eclampsia, n (%)	1 (2.8)	4 (17.4)	0 (0)	*p* = 0.13
Gestational diabetes, n (%)	1 (2.8)	6 (26.1)	1 (33.3)	*p* = 0.02^**§**^
Drugs, n (%)	4 (11.4)	13 (56.5)	1 (33.3)	*p* < 0.0001^**§**^
Antibiotics, n (%)	3 (8.3)	9 (39.1)	1 (33.3)	*p* = 0.01^**§**^
Smoke/illicit drugs, n (%)	0 (0)	0 (0)	0 (0)	*p* > 0.05

^#^ Data available for 36/42 full−term neonates. ^§^ adjusted p−value by the Bonferroni correction for the pairwise comparison between term and preterm non−AKI. *adjusted p−value by the Bonferroni correction for the pairwise comparison between term and preterm non−AKI and between term and preterm AKI. GA: gestational age. AKI: acute kidney injury. IQR: range interquartile. AGA: appropriate for gestational age. LGA: large for gestational age. SGA: small for gestational age. PROM: prolonged rupture of membranes. PIH: pregnancy induced hypertension

### Main outcome

In the cohort of healthy term neonates median uNGAL and TIMP-2*IGFBP7 concentrations were 41.40 (IQR 20.25–74.5) ng/ml e 0.22 (ng/ml)^2^/1000 (IQR 0.14–0.34), respectively.

In the cohort of preterm infants without AKI, uNGAL median concentrations over the three collection times (uNGAL 1, uNGAL 2, and uNGAL 3) were 10.0 ng/ml (IQR 10.0–27.0), 15.75 ng/ml (IQR 10–43.0), and 16.0 ng/ml (IQR 10.0–38.0), respectively. Median concentrations of TIMP-2*IGFBP7 1, TIMP-2*IGFBP7 2, and TIMP-2*IGFBP7 3 were 0.05 (ng/ml)^2^/1000 (IQR 0.04–0.08), 0.08 (ng/ml)^2^/1000 (IQR 0.05–0.24), and 0.06 (ng/ml)^2^/1000 (IQR 0.04–0.15), respectively. For both biomarkers no significant differences between the three time-points values during the first week of life were found (uNGAL *p* = 0.056; TIMP-2*IGFBP7 *p* = 0.15).

**Fig. 1:**
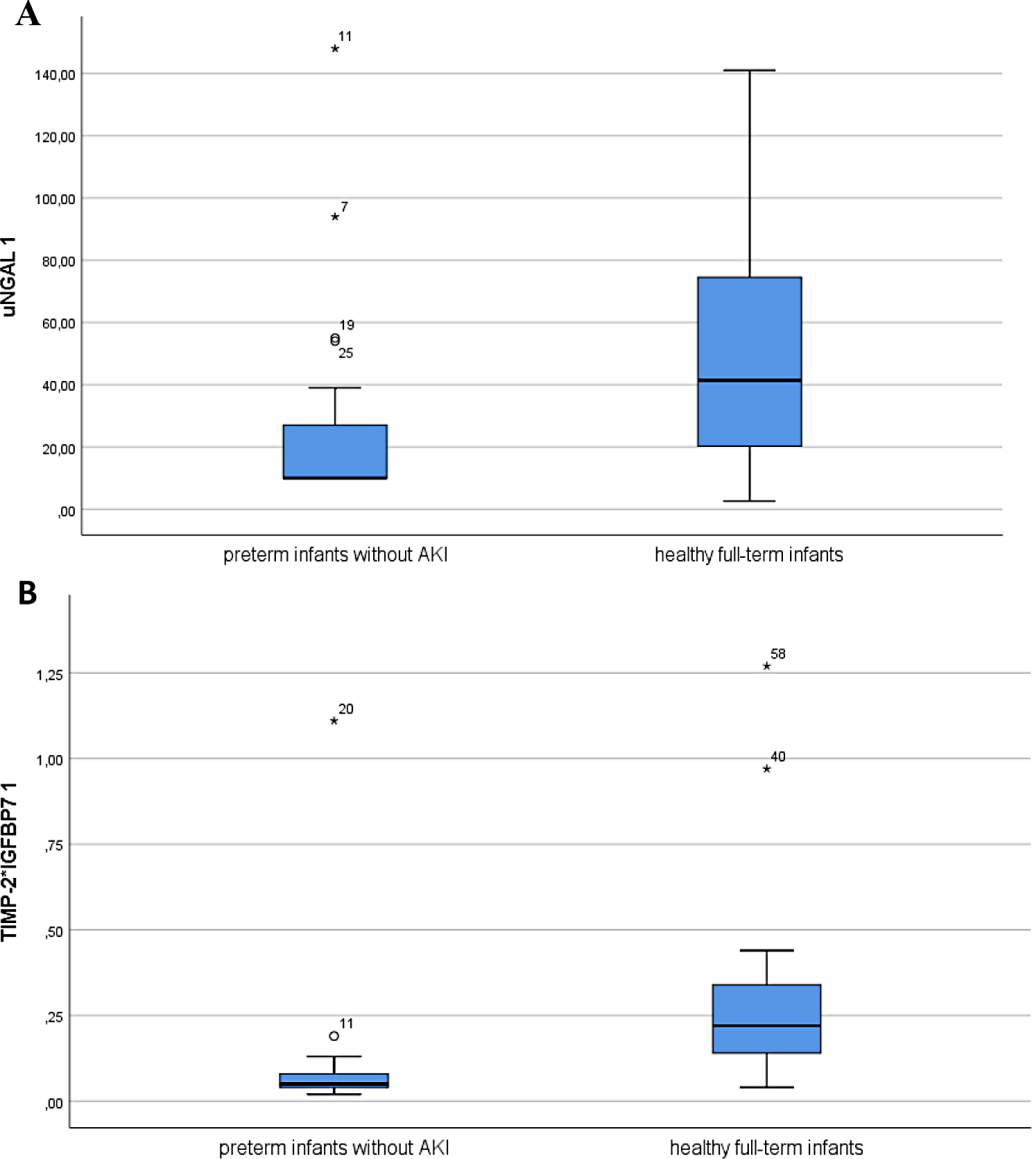
Boxplot comparing median concentrations of uNGAL 1 (**A**) and TIMP-2*IGFBP7 1 (**B**) between preterm infants without AKI and healthy full-term infants

However, median concentration of uNGAL and TIMP-2*IGFBP7 at first collection (DOL 1–3) were significantly lower in preterm infants without AKI than in healthy full-term infants (uNGAL 10.0 vs. 41.4 ng/ml, *p* = 0.007; TIMP-2*IGFBP7 0.05 vs. 0.22 (ng/ml)^2^/1000, *p* < 0.0001) (Fig. 1).

### Correlation with gender, GA and BW in healthy term and preterm infants without AKI

In healthy term neonates no significant differences according to gender were observed both for uNGAL (males 54.0 ng/ml vs. females 41.8 ng/ml) and TIMP-2*IGFBP7 (males 0.23 ng/ml vs. females 0.18 ng/ml) (Mann-Whitney U-test, p  >0.05, for each). Possible factors causing higher uNGAL values or TIMP-2*IGFBP7 were analyzed and the regression analysis showed no significant association between log10-uNGAL values or log10-TIMP-2*IGFBP7 and GA, BW and gender (*p* > 0.05, for each). Spearman’s correlation of uNGAL concentration with TIMP-2*IGFBP7, GA, BW and weight loss revealed a significant relationship only between uNGAL and TIMP-2*IGFBP7 concentrations. Spearman’s rank correlation coefficient (rs) showed a modest positive association between them (rs = 0.40; *p* = 0.009). This finding is confirmed by the linear regression model on log10-transformed data (R^2^ = 0.154, *p* = 0.012).

**Fig. 2:**
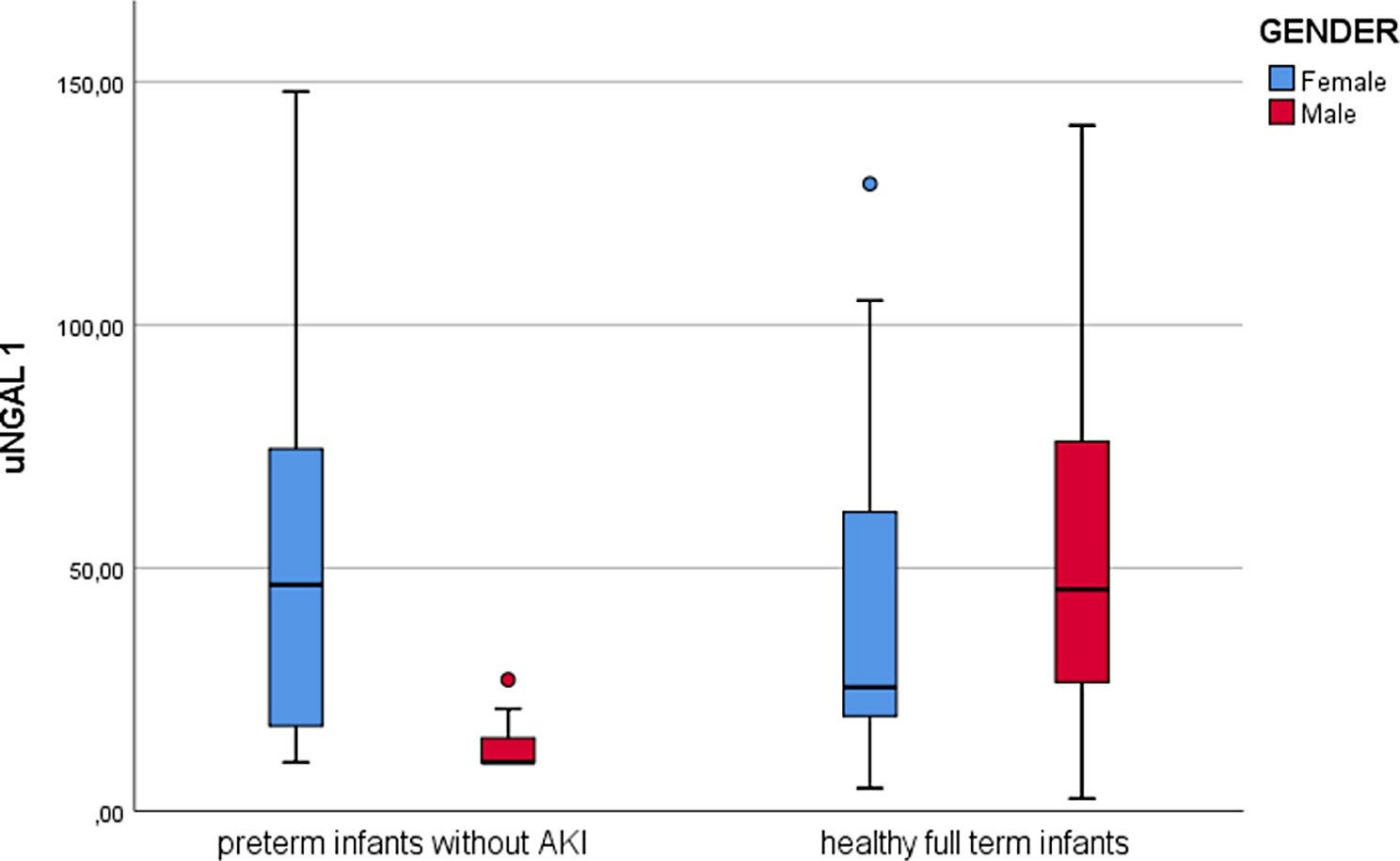
Boxplot comparing median concentrations of uNGAL 1 according to gender between preterm infants without AKI and healthy full-term infants

Similarly, in the cohort of preterm neonates without AKI, the concentrations of uNGAL and TIMP-2*IGFBP7 were not influenced by gender, GA and BW (Table 2), except for higher values of uNGAL in females (46.5 vs. 10.0 ng/ml; Mann-Whitney U-test, *p* = 0.013) (Fig. 2).

**Table 2:** Correlation between gender (A), GA (B) and BW (C) and uNGAL 1 and TIMP-2*IGFBP7 1 in preterm infants without AKI and healthy full-term infants A

A	Preterm non-AKI	Term	*p*-value	
uNGAL 1 • Males, median (IQR) • Females, median (IQR)	10.0 (10.0–15.0) 46.5 (17.5–74.5)	54.0 (29.0–78.0) 41.8 (20.25–85.5)	**0.013**	
TIMP-2*IGFBP7 1 • Males, median (IQR) • Females, median (IQR)	0.05 (0.02–0.08) 0.05 (0.04–0.10)	0.23 (0.15–0.34) 0.18 (0.135–0.325)	0.547	
**B**	**Preterm non-AKI < 32 wks**	**Perterm non-AKI > 32 wks**	**Term**	***p*** **-value**
uNGAL 1, median (IQR)	21.5 (10.0-74.5)	10.0 (10.0–21.0)	41.40 (20.25–74.5)	0.185
TIMP-2*IGFBP7 1, median (IQR)	0.05 (0.03–0.07)	0.05 (0.04–0.09)	0.22 (0.14–0.34)	0.750
**C**	**Preterm non-AKI < 1500 g**	**Preterm non-AKI > 1500 g**	**Term**	***p*** **-value**
uNGAL 1, median (IQR)	16.0 (10.0–41.0)	10.0 (10.0–25.0)	41.40 (20.25–74.5)	0.535
TIMP-2*IGFBP7 1, median (IQR)	0.04 (0.02–0.04)	0.06 (0.04–0.06)	0.22 (0.14–0.34)	0.121

^AKI: acute kidney injury. IQR: interquartile range; wks: weeks^

In the group of preterm without AKI, uNGAL was not different according to GA (< 32 weeks vs. > 32 weeks: 21.5 vs. 10.0 ng/ml; *p* = 0.185) or to BW (< 1500 g vs. > 1500 g: 16.0 vs. 10.0 ng/ml; *p* = 0.535).

Similarly, TIMP-2*IGFBP7 did not change significantly according to sex, GA and BW < 1500 g (*p* > 0.05 for each).

### Accuracy in the diagnosis of AKI in the preterm infant cohort

Applying the modified KDIGO criteria for the neonatal population [7], 3/26 (11.5%) preterm infants developed AKI; two KDIGO stage 1 (7.7%) and one KDIGO stage 2 (3.8%).

Table S1 shows, in preterm neonates with or without AKI, potentially interfering factors with levels of both biomarkers: creatinine (min and max), urinary output and nephrotoxic drugs.

**Fig. 3:**
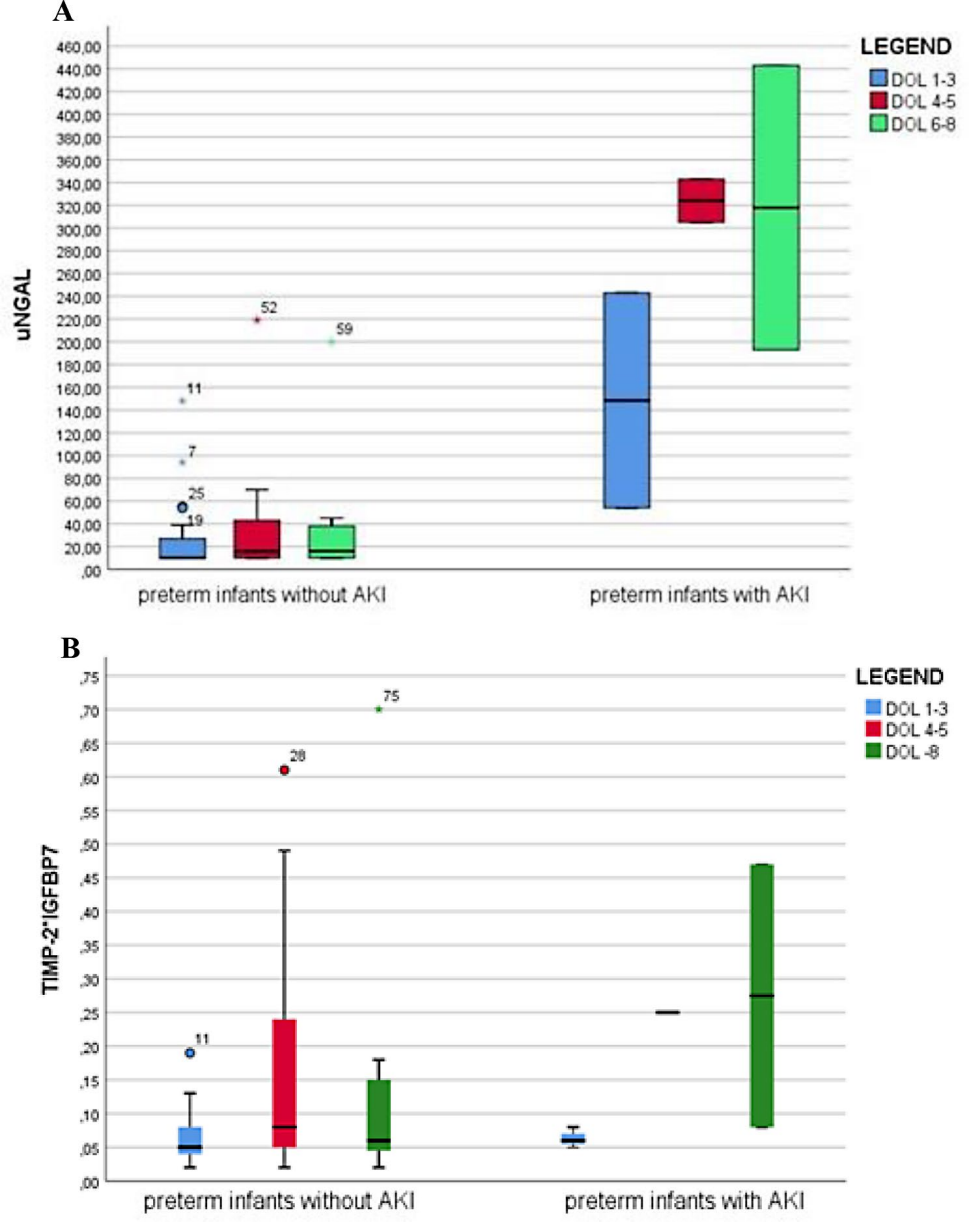
Boxplot of the distribution of uNGAL (**A**) and TIMP-2*IGFBP7 (**B**) in the three collections among preterm infants with and without AKI

In preterm infants who developed AKI during the first week of life uNGAL in all three time-points was higher compared to preterm infants without AKI (148.5 vs. 10.0, *p* = 0.04; 324.0 vs. 15.75, *p* = 0.02; 318.0 vs. 16.0 ng/ml, *p* = 0.04) (Fig. 3A), while TIMP-2*IGFBP7 were not different between preterm infants with or without AKI (0.06 vs. 0.05; 0.08 vs. 0.08; 0.27 vs. 0.06 (ng/ml)^2^/1000, *p* > 0.05) (Fig. 3B).

The use of nephrotoxic drugs did not significantly affect the values of uNGAL and TIMP-2*IGFBP7 in the three evaluations, despite a slight increase in the urinary levels of the two biomarkers at the second time-point (data not shown).

No neonates had sepsis during the first week of life. However, those who developed AKI in the first week of life had longer and more critical clinical courses, with increased need and duration of ventilatory support. 

Secondary outcomes are described in Table S2.

The diagnostic accuracy of uNGAL and TIMP-2*IGFBP7 in preterm infants who developed AKI during the first week of life was evaluate and the ROC curve of uNGAL1 as a predictor of AKI showed an area under the curve (AUC) of 0.92 (*p* = 0.024) with an optimal cut-off of 46.5 ng/ml (81%; 100%), while the ROC curve of TIMP-2*IGFBP7 1 showed an AUC of 0.59 (*p* = 0.330) with an optimal cut-off of 0.045 (ng/ml)^2^/1000 (38.1%; 100%).

## Discussion

AKI affects 18–70% of neonates admitted to NICUs [22–29]. The significant incidence of AKI in neonates (especially in very and extremely low birth weight infants) is due to both maternal and neonatal risk factors interfering with the dynamic physiology of the neonatal kidney [30]. AKI incidence in our study (11.5%) is lower than previous studies, though similar to data reported by Chen et al. [31], but this is due to the exclusion of newborns with critical clinical course.

Significant efforts have been made to identify markers for early diagnosis and prediction of AKI. Since in newborns both sCr and UOP are not optimal markers of AKI, several novel biomarkers gained attention, as well as uNGAL and TIMP-2*IGFBP7, but the lack of established range values have limited their widespread use.

Our study shows a median uNGAL concentration of 41.40 (IQR 20.25–74.5) ng/ml and a median TIMP-2*IGFBP7 of 0.22 (ng/ml)^2^/1000 (IQR 0.14–0.34) in healthy term infants. Previous studies showed a median uNGAL concentration in term neonates ranging from 6.8 to 88.1 ng/ml [32–38] and a median TIMP-2*IGFBP7 of 0.13 [19]. In preterm infants without AKI, uNGAL median concentrations over the three collection times (uNGAL 1, uNGAL 2, and uNGAL 3) were 10.0 ng/ml (IQR 10.0–27.0), 15.75 ng/ml (IQR 10–43.0), and 16.0 ng/ml (IQR 10.0–38.0), respectively. These results were similar to those reported by Huynh et al. (5 ng/ml) in 50 neonates with median GA of 29 weeks *±* 1.6 and without renal injury [39]. Conversely, our results are lower than other studies reporting values from 41.52 to 424 ng/ml [34, 40–43]. In the same cohort, median concentrations of TIMP-2*IGFBP7 1, TIMP-2*IGFBP7 2, and TIMP-2*IGFBP7 3 were 0.05 (ng/ml)^2^/1000 (IQR 0.04–0.08), 0.08 (ng/ml)^2^/1000 (IQR 0.05–0.24), and 0.06 (ng/ml)^2^/1000 (IQR 0.04–0.15), respectively, similar to data by Chen et al. [31].

No statistically significant differences between full-term males and females were observed. Previous studies showed that uNGAL levels are also not influenced by GA and birth weight [33, 44], whereas this information is still lacking for TIMP-2*IGFBP7. However, some authors reported significantly higher uNGAL values in female neonates, probably due to different measurement methods (CMIA vs. ELISA), reagents or antibodies [34, 37].

In our study preterm female neonates without AKI have higher uNGAL than male neonates (46.5 vs. 10.0 ng/ml; Mann-Whitney U-test, *p* =0.013), confirming the influence of gender on this urinary biomarker as other reported [39, 42, 43]. No differences according to gender were observed for the median concentration of TIMP-2*IGFBP7, in contrast with Chen et al. [31] who showed higher values in male newborns.

In our study, both uNGAL and TIMP-2*IGFBP7 levels in preterm neonates without AKI were lower compared to healthy term infants (*p* = 0.007 e *p* < 0.0001, respectively) and not influenced by GA and BW, whereas previous studies showed an inverse relationship between uNGAL and GA or BW [39, 43, 45, 46].

In the present study, 3/26 (11.5%) preterm infants developed AKI according to the modified KDIGO criteria for the neonatal population [7]. In these newborns uNGAL increased quickly and predicts the development of AKI, with an AUC of 0.92. These results are in accordance with recently published data by Hanna et al. [47]. The AUC of TIMP-2*IGFBP7 was instead low (0.59) and its role for AKI prediction is not evident, differently from Chen study [31].

Our findings confirm that uNGAL is among the most promising biomarkers of AKI: it is rapidly synthesized and released from the damaged distal nephron in experimental disease models; it is easily detectable both in plasma and in urine, irrespective of other biological variables (unlike sCr); its concentrations increase according to severity and duration of kidney injury and decrease rapidly once the insult is over [13]; it is useful for early diagnosis of AKI and prediction of clinical outcome (need for dialysis and mortality risk). uNGAL could be used for early diagnosis of AKI as well as a biomarker of kidney damage when nephrotoxic drugs are used [48]. NGAL is an effective early biomarker of AKI in a wide range of neonatal and pediatric intensive care scenarios (e.g. perinatal asphyxia [49], cardiac surgery [50]) predicting renal complications with good sensitivity as early as two days before sCr increase [51, 52].

In recent years, TIMP-2*IGFBP7 proved effective in predicting AKI in the critical adult [15, 18]. Two different cut-off values (0.3 and 2.0 ng/ml^2^/1000) of TIMP-2*IGFBP7 were validated in critically-ill adult patients [14, 53], showing that patients with TIMP-2*IGFBP7 value > 0.3 had a seven-fold increase in AKI risk [14] and that the risk of death, dialysis or recurrent renal dysfunction in patients with AKI was doubled for values above 2.0 [15].

However, whether TIMP-2*IGFBP7 can be routinely used in pediatric and neonatal settings remains an open question. The performance of TIMP-2*IGFBP7 in pediatric patients was evaluated by Westhoff et al. [19] on a cohort of 133 patients aged 0–18 years, of which 46 with AKI according to pediatric Risk, Injury, Failure, Loss, End Stage Renal Disease (pRIFLE) criteria, 27 without AKI (non-AKI group I) and 60 apparently healthy neonates and children (non-AKI group II). Patients in the “Failure” stage showed a median 3.7-fold higher urinary TIMP-2*IGFBP7 compared to non-AKI subjects (*p* < 0.001). TIMP-2*IGFBP7 had a significant chance to predict mortality after 30 days and 3 months, and moderate role in predicting renal replacement therapy (RRT). According to their findings, TIMP-2*IGFBP7 median values in the two different control groups (0.10 and 0.27), including both neonates and children, were largely stable and comparable to adults, but with a tendency to be lower in neonates and younger children. In our study TIMP-2*IGFBP7 median values are similar to those reported by Westhoff et al. in their control groups [19]. Urinary TIMP-2*IGFBP7 proved effective for the early diagnosis of neonatal AKI in 14 indomethacin-treated very low birth weight (VLBW) infants [54] and in 31 infants after cardiac surgery in which TIMP-2*IGFBP7 values ≥ 0.78 predicted serum increase of milrinone concentration prior to serum creatinine [55]. Meersch et al. found that pediatric patients with congenital heart disease had elevated urinary TIMP-2*IGFBP7 values before cardiac surgery (mean 0.9–1.0) with values decreasing to 0.4–0.5 one day after surgery, suggesting that either preoperative venous congestion or fasting could affect renal integrity [56]. However, more recently, Bojan et al. discussed the usefulness of TIMP-2*IGFBP7 for the prediction of cardiac surgery-related AKI in neonates and infants when measured within 3 h of cardiopulmonary bypass [57]. Accordingly to their study, we also showed a lower efficacy of TIMP-2*IGFBP7 for the early detection of neonatal AKI, as shown by a low AUC (0.59).

### Strengths and limitations

To the best of our knowledge, this is the first study to focus on uNGAL and TIMP-2*IGFBP7 values in healthy term and preterm neonates. Our study highlights also that in clinically stable preterm neonates, AKI may occur.

We acknowledge some limitations of the study. Firstly, the small sample of patients may impact the reliability and reproducibility of findings. Secondly, we did not use uNGAL/creatinine ratio to assess renal function. Finally, both urinary NGAL and TIMP-2*IGFBP7 in healthy full-term infants were assessed only once per patient so providing only a static picture of normal range values for urinary NGAL and TIMP-2*IGFBP7 in this cohort, missing the possible dynamic evolution of these biomarkers over time.

### Future directions

The future direction for urinary AKI biomarkers implementation in neonates is likely to be a dynamic and multidimensional approach, as recently suggested by Basu [58]. An “AKI Biomarker Composite” (ABC) panel over time may improve the recognition and management of AKI phenotype, according to stage and subtype (e.g. tubular, glomerular, reduced compensation, increased system stress…). That would be an important step towards tailored medicine, possibly combined with the determination of the individual genomic profile. Indeed, precision laboratory diagnostics allows to inform treatment choices and reduce the use of invasive procedures for conditions with high impact on newborns' health and quality of life like AKI [59].

## Conclusions

AKI in neonates continues to be an underestimated clinical issue. Since sCr and UOP are inaccurate markers of renal function, novel urinary biomarkers such as uNGAL and TIMP-2*IGFBP7 may be helpful for the early recognition and treatment of neonates at risk for mild to severe AKI, also because they are non-invasive determinations. The influence of factors such as gender, gestational age and birth weight may require more data to confirm possible relationships. Our study shows that uNGAL could be a more useful marker of early stage of AKI than TIMP-2*IGFBP7, especially in preterm neonates. Further data are needed to confirm its diagnostic role and to evaluate the role of both biomarkers during AKI, focusing on their changes due to the treatment and the evolution of this disease in the very vulnerable population of neonates.

## Electronic supplementary material

Below is the link to the electronic supplementary material.

Supplementary Material 1

Supplementary Material 2

Supplementary Material 3

## Data Availability

The datasets generated and analysed during the current study are available from the corresponding author on reasonable request.

## Abbreviations

AGA: Appropriate for gestational age
AKI: Acute Kidney Injury
AUC: Area under the curve
AWAKEN: Assessment of Worldwide Acute Kidney Injury Epidemiology in Neonates
BPD: Bronchopulmonary dysplasia
BW: Birth weight
CAKUT: Congenital anomalies of kidney and urinary tract
CV%: Imprecision
DOL: Days of life
FDA: Food and Drug Administration
GA: Gestational age
GFR: Glomerular filtration rate
IGFBP: Insulin-like Growth Factor-Binding Protein
IGFBP7: Insulin-like Growth Factor-Binding Protein 7
IGFs: Insulin-like Growth Factors
IQR: Interquartile range
IVH: Intraventricular hemorrhage
KDIGO: Kidney Disease Improving Global Outcomes
LGA: Large for gestational age
LoD: Limit of detection
MMP: Matrix metalloproteinase
NEC: Necrotizing enterocolitis
NGAL: Neutrophil Gelatinase-associated Lipocalin
NICUs: Neonatal intensive care units
PDA: Patent ductus arteriosus
PIH: Pregnancy induced hypertension
pRIFLE: Pediatric Risk, Injury, Failure, Loss, End Stage Renal Disease
PROM: Prolonged rupture of membranes
PVL: Periventricular leukomalacia
ROP: Retinopathy of prematurity
RRT: Renal replacement therapy
rs: Spearman’s rank correlation coefficient
sCr: Serum creatinine
SD: Standard deviation
SGA: Small for gestational age
STROBE: STrengthening the Reporting of OBservational studies in Epidemiology
TIMP-2: Tissue Inhibitor of Metalloproteinase-2
uNGAL: Urinary Neutrophil Gelatinase-associated Lipocalin
UOP: Urinary output
VLBW: Very low birth weight
wks: Weeks

## References

[1] Slater MB, Anand V, Uleryk EM, Parshuram CS. A systematic review of RIFLE criteria in children, and its application and association with measures of mortality and morbidity. Kidney Int. 2012;81:791–8. 22258324 10.1038/ki.2011.466

[2] Srisawat N, Hoste EEA, Kellum JA. Modern classification of acute kidney injury. Blood Purif. 2010;29:300–7. 20130395 10.1159/000280099

[3] Jetton JG, Boohaker LJ, Sethi SK, et al. Incidence and outcomes of neonatal acute kidney injury (AWAKEN): a multicentre, multinational, observational cohort study. Lancet Child Adolesc Health. 2017;1:184–94. 29732396 10.1016/S2352-4642(17)30069-X PMC5933049

[4] Selewski DT, Charlton JR, Jetton JG, et al. Neonatal acute kidney injury. Pediatrics. 2015;136:e463–73. 26169430 10.1542/peds.2014-3819

[5] Gallo D, de Bijl-Marcus KA, Alderliesten T, Lilien M, Groenendaal F. Early acute kidney injury in preterm and term neonates: incidence, outcome, and Associated Clinical features. Neonatology. 2021;118(2):174–9. 10.1159/000513666 33780939

[6] Auron A, Mhanna MJ. Serum creatinine in very low birth weight infants during their first days of life. J Perinatol. 2006;26:755–60. 17036033 10.1038/sj.jp.7211604

[7] Kellum JA, Lameire N, Aspelin P et al. Kidney disease: Improving global outcomes (KDIGO) acute kidney injury work group. KDIGO clinical practice guideline for acute kidney injury. Kidney Int Suppl (2011). 2012;2:1–138.

[8] Greenberg JH, Parikh CR. Biomarkers for diagnosis and prognosis of AKI in Children: one size does not fit all. Clin J Am Soc Nephrol. 2017;12:1551–7. 28667085 10.2215/CJN.12851216 PMC5586584

[9] Drukker A, Guignard J-P. Renal aspects of the term and preterm infant: a selective update. Curr Opin Pediatr. 2002;14:175–82. 11981287 10.1097/00008480-200204000-00006

[10] Allegaert K, Smits A, van Donge T, et al. Renal Precision Medicine in neonates and Acute kidney Injury: how to convert a cloud of Creatinine observations to support clinical decisions. Front Pediatr. 2020;8:366. 32850523 10.3389/fped.2020.00366 PMC7399072

[11] Oddie S, Adappa R, Wyllie J. Measurement of urine output by weighing nappies. Arch Dis Child Fetal Neonatal Ed. 2004;89:F180–1. 14977908 10.1136/adc.2002.018853 PMC1756039

[12] Fernández-Ruiz M, Calvo B, Vara R, Villar RN, Aguado JM. Inappropriate use of urinary catheters in patients admitted to medical wards in auniversity hospital. Enferm Infecc Microbiol Clin. 2013;31:523–5. 23601704 10.1016/j.eimc.2013.02.013

[13] Sarafidis K, Tsepkentzi E, Diamanti E, et al. Urine neutrophil gelatinase-associated lipocalin to predict acute kidney injury in preterm neonates. A pilot study. Pediatr Nephrol. 2014;29:305–10. 24022367 10.1007/s00467-013-2613-6

[14] Bihorac A, Chawla LS, Shaw AD, et al. Validation of cell-cycle arrest biomarkers for acute kidney injury using clinical adjudication. Am J Respir Crit Care Med. 2014;189:932–9. 24559465 10.1164/rccm.201401-0077OC

[15] Kashani K, Al-Khafaji A, Ardiles T, et al. Discovery and validation of cell cycle arrest biomarkers in human acute kidney injury. Crit Care. 2013;17:R25. 23388612 10.1186/cc12503 PMC4057242

[16] Goetz DH, Holmes MA, Borregaard N, Bluhm ME, Raymond KN, Strong RK. The Neutrophil Lipocalin NGAL is a Bacteriostatic Agent that interferes with siderophore-mediated Iron Acquisition. Mol Cell. 2002;10:1033–43. 12453412 10.1016/s1097-2765(02)00708-6

[17] Vijayan A, Faubel S, Askenazi DJ, et al. Clinical use of the urine biomarker [TIMP-2] × [IGFBP7] for acute kidney Injury Risk Assessment. Am J Kidney Dis. 2016;68:19–28. 26948834 10.1053/j.ajkd.2015.12.033 PMC4921267

[18] Fan W, Ankawi G, Zhang J, et al. Current understanding and future directions in the application of TIMP-2 and IGFBP7 in AKI clinical practice. Clin Chem Lab Med. 2019;57:567–76. 30179848 10.1515/cclm-2018-0776

[19] Westhoff JH, Tönshoff B, Waldherr S, et al. Urinary tissue inhibitor of Metalloproteinase-2 (TIMP-2) • insulin-like Growth factor-binding protein 7 (IGFBP7) predicts adverse outcome in Pediatric Acute kidney Injury. PLoS ONE. 2015;10:e0143628. 26606754 10.1371/journal.pone.0143628 PMC4659607

[20] von Elm E, Altman DG, Egger M, et al. Strengthening the reporting of Observational studies in Epidemiology (STROBE) statement: guidelines for reporting observational studies. BMJ. 2007;335:806–8. 17947786 10.1136/bmj.39335.541782.AD PMC2034723

[21] Nalesso F, Cattarin L, Gobbi L, Fragasso A, Garzotto F, Calò LA. Evaluating Nephrocheck^®^ as a Predictive Tool for Acute kidney Injury. Int J Nephrol Renovasc Dis. 2020;13:85–96. 32425580 10.2147/IJNRD.S198222 PMC7189184

[22] Blinder JJ, Asaro LA, Wypij D, et al. Acute kidney Injury after Pediatric Cardiac surgery: a secondary analysis of the Safe Pediatric Euglycemia after cardiac surgery trial. Pediatr Crit Care Med. 2017;18:638–46. 28492399 10.1097/PCC.0000000000001185 PMC5503840

[23] Gadepalli SK, Selewski DT, Drongowski RA, Mychaliska GB. Acute kidney injury in congenital diaphragmatic hernia requiring extracorporeal life support: an insidious problem. J Pediatr Surg. 2011;46:630–5. 21496529 10.1016/j.jpedsurg.2010.11.031

[24] Kaur S, Jain S, Saha A, et al. Evaluation of glomerular and tubular renal function in neonates with birth asphyxia. Ann Trop Paediatr. 2011;31:129–34. 21575317 10.1179/146532811X12925735813922

[25] Koralkar R, Ambalavanan N, Levitan EB, McGwin G, Goldstein S, Askenazi D. Acute kidney injury reduces survival in very low birth weight infants. Pediatr Res. 2011;69:354–8. 21178824 10.1203/PDR.0b013e31820b95ca

[26] Sarkar S, Askenazi DJ, Jordan BK, et al. Relationship between acute kidney injury and brain MRI findings in asphyxiated newborns after therapeutic hypothermia. Pediatr Res. 2014;75:431–5. 24296799 10.1038/pr.2013.230

[27] Selewski DT, Jordan BK, Askenazi DJ, Dechert RE, Sarkar S. Acute kidney injury in asphyxiated newborns treated with therapeutic hypothermia. J Pediatr. 2013;162:725–e7291. 23149172 10.1016/j.jpeds.2012.10.002

[28] Carmody JB, Swanson JR, Rhone ET, Charlton JR. Recognition and reporting of AKI in very low birth weight infants. Clin J Am Soc Nephrol. 2014;9:2036–43. 25280497 10.2215/CJN.05190514 PMC4255405

[29] Charlton JR, Boohaker L, Askenazi D, et al. Incidence and risk factors of early onset neonatal AKI. Clin J Am Soc Nephrol. 2019;14:184–95. 31738181 10.2215/CJN.03670318 PMC6390916

[30] Harer MW, Selewski DT, Kashani K et al. Improving the quality of neonatal acute kidney injury care: neonatal-specific response to the 22nd Acute Disease Quality Initiative (ADQI) conference. Journal of Perinatology. 2020. 10.1038/s41372-020-00810-z 32892210

[31] Chen J, Sun Y, Wang S, et al. The effectiveness of urinary TIMP-2 and IGFBP-7 in predicting acute kidney injury in critically ill neonates. Pediatr Res. 2020;87:1052–9. 31791043 10.1038/s41390-019-0698-8

[32] Cangemi G, Storti S, Cantinotti M, et al. Reference values for urinary neutrophil gelatinase-associated lipocalin (NGAL) in pediatric age measured with a fully automated chemiluminescent platform. Clin Chem Lab Med. 2013;51:1101–5. 23183760 10.1515/cclm-2012-0540

[33] Mikulić V, Rogić D, Mikulić I, et al. Urine neutrophil gelatinase-associated lipocalin concentration in healthy newborns during the first three postnatal days. Biochem Med (Zagreb). 2020;30:1–5. 10.11613/BM.2020.030706 PMC7528642 33071557

[34] Chen C-N, Chou C-H, Jeng S-F, et al. Urinary Neutrophil Gelatinase-Associated Lipocalin levels in neonates. Pediatr Neonatol. 2016;57:207–12. 26563762 10.1016/j.pedneo.2015.09.003

[35] Sarafidis K, Tsepkentzi E, Agakidou E, et al. Serum and urine acute kidney injury biomarkers in asphyxiated neonates. Pediatr Nephrol. 2012;27:1575–82. 22532328 10.1007/s00467-012-2162-4

[36] Abdelhady S, Gawad ERA, Haie OMA, Mansour AI. Usefulness of serum and urinary Neutrophil Gelatinase -Associated Lipocalin in detecting Acute kidney Injury in Asphyxiated neonates. Int J Med Health Sci. 2016;5:230–6.

[37] Kamianowska M, Wasilewska A, Szczepański M, Kulikowska E, Bebko B, Koput A. Health term-born girls had higher levels of urine neutrophil gelatinase-associated lipocalin than boys during the first postnatal days. Acta Paediatr. 2016;105:1105–8. 27359090 10.1111/apa.13508

[38] Krzeminska E, Wyczalkowska-Tomasik A, Korytowska N, Paczek L. Comparison of two methods for determination of NGAL levels in urine: ELISA and CMIA. J Clin Lab Anal. 2016;30:956–60. 27075972 10.1002/jcla.21962 PMC6806694

[39] Huynh TK, Bateman DA, Parravicini E, et al. Reference values of urinary neutrophil gelatinase-associated lipocalin in very low birth weight infants. Pediatr Res. 2009;66:528–32. 19680166 10.1203/PDR.0b013e3181baa3dd PMC3482111

[40] DeFreitas MJ, Seeherunvong W, Katsoufis CP, et al. Longitudinal patterns of urine biomarkers in infants across gestational ages. Pediatr Nephrol. 2016;31:1179–88. 26862052 10.1007/s00467-016-3327-3

[41] De Mul A, Parvex P, Wilhelm-Bals A. Neutrophil gelatinase-associated lipocalin distribution in preterm newborns without acute kidney injury as defined by a reference method. J Matern Fetal Neonatal Med. 2022;35(25):4956–60. 10.1080/14767058.2021.1873939 33455508

[42] Suchojad A, Tarko A, Smertka M, et al. Factors limiting usefulness of serum and urinary NGAL as a marker of acute kidney injury in preterm newborns. Ren Fail. 2015;37:439–45. 25598237 10.3109/0886022X.2014.996109

[43] Lavery AP, Meinzen-Derr JK, Anderson E, et al. Urinary NGAL in premature infants. Pediatr Res. 2008;64:423–8. 18552711 10.1203/PDR.0b013e318181b3b2

[44] Elmas AT, Tabel Y, Ipek S. Determination of reference values for urinary neutrophil gelatinase-associated lipocalin in premature infants. J Maternal-Fetal Neonatal Med. 2014;27:187–91. 10.3109/14767058.2013.806900 23682838

[45] Saeidi B, Koralkar R, Griffin RL, Halloran B, Ambalavanan N, Askenazi D. Impact of gestational age, sex, and postnatal age on urine biomarkers in premature neonates. Pediatr Nephrol. 2015;30:2037. 26001700 10.1007/s00467-015-3129-z PMC4581905

[46] Askenazi DJ, Koralkar R, Levitan EB, et al. Baseline values of candidate urine acute kidney injury biomarkers vary by gestational age in premature infants. Pediatr Res. 2011;70:302–6. 21646940 10.1203/PDR.0b013e3182275164 PMC3152663

[47] Hanna M, Brophy PD, Giannone PJ, Joshi MS, Bauer JA, RamachandraRao S. Early urinary biomarkers of acute kidney injury in preterm infants. Pediatr Res 2016. 2016;80:2. 10.1038/pr.2016.70 27055185

[48] Haase-Fielitz A, Bellomo R, Devarajan P, et al. The predictive performance of plasma neutrophil gelatinase-associated lipocalin (NGAL) increases with grade of acute kidney injury. Nephrol Dial Transpl. 2009;24:3349–54. 10.1093/ndt/gfp234 19474273

[49] Bellos I, Fitrou G, Daskalakis G, Perrea DN, Pergialiotis V. Neutrophil gelatinase-associated lipocalin as predictor of acute kidney injury in neonates with perinatal asphyxia: a systematic review and meta-analysis. Eur J Pediatr. 2018;177:1425–34. 30051145 10.1007/s00431-018-3221-z

[50] Parikh CR, Devarajan P, Zappitelli M, et al. Postoperative biomarkers predict acute kidney injury and poor outcomes after pediatric cardiac surgery. J Am Soc Nephrol. 2011;22:1737–47. 21836147 10.1681/ASN.2010111163 PMC3171944

[51] Zappitelli M, Washburn KK, Arikan AA, et al. Urine neutrophil gelatinase-associated lipocalin is an early marker of acute kidney injury in critically ill children: a prospective cohort study. Crit Care. 2007;11:R84. 17678545 10.1186/cc6089 PMC2206519

[52] Wheeler DS, Devarajan P, Ma Q, et al. Serum neutrophil gelatinase-associated lipocalin (NGAL) as a marker of acute kidney injury in critically ill children with septic shock. Crit Care Med. 2008;36:1297–303. 18379258 10.1097/CCM.0b013e318169245a PMC2757115

[53] Hoste EAJ, McCullough PA, Kashani K, et al. Derivation and validation of cutoffs for clinical use of cell cycle arrest biomarkers. Nephrol Dial Transpl. 2014;29:2054–61. 10.1093/ndt/gfu292 PMC4209880 25237065

[54] Waldherr S, Fichtner A, Beedgen B, et al. Urinary acute kidney injury biomarkers in very low-birth-weight infants on indomethacin for patent ductus arteriosus. Pediatr Res. 2019;85:678–86. 30745571 10.1038/s41390-019-0332-9

[55] Gist KM, Cooper DS, Wrona J, et al. Acute kidney Injury biomarkers predict an increase in serum Milrinone Concentration earlier than serum creatinine-defined acute kidney Injury in infants after cardiac surgery. Ther Drug Monit. 2018;40:186–94. 29529007 10.1097/FTD.0000000000000496 PMC5851490

[56] Meersch M, Schmidt C, Van Aken H, et al. Validation of cell-cycle arrest biomarkers for acute kidney injury after pediatric cardiac surgery. PLoS ONE. 2014;9:e110865. 25343505 10.1371/journal.pone.0110865 PMC4208780

[57] Bojan M, Pieroni L, Semeraro M, Froissart M. Cell-cycle arrest biomarkers: usefulness for cardiac surgery-related acute kidney Injury in neonates and Infants*. Pediatr Crit Care Med. 2020;21:563–70. 32195906 10.1097/PCC.0000000000002270

[58] Basu RK. Dynamic Biomarker Assessment: a diagnostic paradigm to Match the AKI Syndrome. Front Pediatr. 2020;7:535. 32039106 10.3389/fped.2019.00535 PMC6986245

[59] Serra G, Corsello G, Antona V, et al. Autosomal recessive polycystic kidney disease: case report of a newborn with rare PKHD1 mutation, rapid renal enlargement and early fatal outcome. Ital J Pediatr. 2020;46:154. 33059727 10.1186/s13052-020-00922-4 PMC7560064

