# Supplementary material for: Evaluation of uNGAL and TIMP-2*IGFBP7 as early biomarkers of Acute Kidney Injury in Caucasian term and preterm neonates: a prospective observational cohort study
Source: Ital J Pediatr. 2025 Mar 1;51:64. doi: 10.1186/s13052-025-01899-8 (PMC11872328; doi:10.1186/s13052-025-01899-8)
Supplement: Supplementary file 1 — Supplementary Material 1 [file 13052_2025_1899_MOESM1_ESM.docx]

**Figure S1: STROBE Flow diagram for full-term healthy (A) and preterm (B) infants**

**A.**

Enrolled patients (n=112)

## Inclusion

Included in the study (n=44)

## Analysis

Analysed (n=42)

## Enrollment

Excluded (n=68)

♦  Outborn (n=11)

♦  Comorbidities (n=19)

♦  Failure to collect urine sample (n=38)

Excluded (n=2)

♦  Outliers (n=2)

**B.**

## Enrollment

Enrolled patients (n=105)

Included in the study (n= 27)

Excluded (n=78)

- Malformations or major comorbidities (n=11)
- Failure to collect urine sample (n=11)
- Missing informed consent (n=3)
- Outborn admitted after 72 hours (n=6)
- Admission <8 days (n=47)

## Inclusion

Excluded (n= 1)

- Insufficient data (n=1)

Analysed (n=26)

- Non-AKI* (n=23)
- AKI* (n=3)

## Analysis

**according to KDIGO modified criteria for the neonatal population*
