# Supplementary material for: Evaluation of uNGAL and TIMP-2*IGFBP7 as early biomarkers of Acute Kidney Injury in Caucasian term and preterm neonates: a prospective observational cohort study
Source: Ital J Pediatr. 2025 Mar 1;51:64. doi: 10.1186/s13052-025-01899-8 (PMC11872328; doi:10.1186/s13052-025-01899-8)
Supplement: Supplementary file 2 — Supplementary Material 2 [file 13052_2025_1899_MOESM2_ESM.docx]

|  | **Preterm total**  **n = 26** | **Preterm non-AKI**  **n=23** | **Preterm AKI**  **n=3** | **p-value** |
| --- | --- | --- | --- | --- |
| Creatinine min (mg/dl),  median (IQR) | 0.76  (0.70-0.84) | 0.78  (0.71-0.85) | 0.42  (0.33-0.59) | 0.066 |
| Creatinine max (mg/dl),  median (IQR) | 0.85  (0.71-1.13) | 0.76  (0.67-0.85) | 0.93  (0.84-1.13) | 0.40 |
| Diuresis (ml/kg/h),  median (IQR) | 2.04  (1.80-2.77) | 2.00  (1.80-2.69) | 2.80  (2.10-3.25) | 0.648 |
| Nephrotoxic drugs,  n (%) | 21 (80.8) | 18 (78.3) | 3 (100) | 1.00 |

**Table S1:** **Factors potentially influencing uNGAL and TIMP-2*IGFBP7 in the cohort of preterm infants with and without diagnosis of AKI**

^AKI: acute kidney injury. IQR: interquartile range.^
