# Supplementary material for: Evaluation of uNGAL and TIMP-2*IGFBP7 as early biomarkers of Acute Kidney Injury in Caucasian term and preterm neonates: a prospective observational cohort study
Source: Ital J Pediatr. 2025 Mar 1;51:64. doi: 10.1186/s13052-025-01899-8 (PMC11872328; doi:10.1186/s13052-025-01899-8)
Supplement: Supplementary file 3 — Supplementary Material 3 [file 13052_2025_1899_MOESM3_ESM.docx]

**Table S2: Secondary outcomes**

|  | **Preterm total**  **n = 26** | **Preterm non-AKI n=23** | **Preterm AKI**  **n=3** | **p-value** |
| --- | --- | --- | --- | --- |
| Invasive ventilation, n (%) | 3 (12) | 1 (4.5) | 2 (66.7) | **0.029** |
| BPD 28 days, n (%) | 4 (15.4) | 2 (8.7) | 2 (66.7) | 0.052 |
| BPD 36 weeks, n (%) | 2 (7.7) | 1 (4.3) | 1 (33.3) | 2.222 |
| Air leak, n (%) | 0 (0) | 0 (0) | 0 (0) | n.a. |
| Days of ventilatory support, median (IQR) | 2  (0.25-5.75) | 1.00  (0.00-4.00) | 52.00  (37.00-89.00) | **0.003** |
| Days of invasive ventilation, median (IQR) | 0.00  (0.00-0.00) | 0.00  (0.00-0.00) | 16.00  (8.00-16.50) | 0.078 |
| Days of non-invasive ventilation, median (IQR) | 2.00  (0.25-5.75) | 1.00  (0.00-4.00) | 35.00  (28.50-72.50) | **0.005** |
| Postnatal steroids, n (%) | 5 (19.2) | 3 (13.0) | 2 (66.7) | 0.085 |
| Sepsis 1st week, n (%) | 0 (0) | 0 (0) | 0 (0) | n.a. |
| Sepsis after 1st week,  n (%) | 4 (15.4) | 2 (8.7) | 2 (66.7) | 0.052 |
| PDA, n (%) | 6 (23.1) | 3 (13.0) | 3 (100) | **0.008** |
| PDA   - no treatment - ibuprofen - paracetamol - both - surgery | 3 (11.5)  0 (0)  0 (0)  3 (11.5)  0 (0) | 3 (13.0)  0 (0)  0 (0)  0 (0)  0 (0) | 0 (0)  0 (0)  0 (0)  3 (100)  0 (0) | **0.001** |
| ROP stage 3-4 or requiring laser, n (%) | 2 (7.7) | 0 (0) | 2 (66.7) | **0.009** |
| NEC stage > 2, n (%)   - medical treatment - surgery | 0 (0)  1 (3.8) | 0 (0)  0 (0) | 0 (0)  1 (33.3) | 0.120 |
| Intestinal perforation,  n (%) | 1 (3.8) | 0 (0) | 1 (33.3) | 0.115 |
| crUSS pathological findings,  n (%)   - IVH 3-4 grade - PVL - Post-haemorragic hydrocephalus | 0 (0)  0 (0)  1 (3.8) | 0 (0)  0 (0)  0 (0) | 0 (0)  0 (0)  1 (33.3) | 0.115 |
| Weight at discharge,  median (IQR) | 2022.50 (1891.25-2226.25) | 1995.00 (1867.50-2165.50) | 2835.00 (2440.00-2882.50) | **0.032** |
| GA at discharge,  median (IQR) | 35.86  (35.00-36.82) | 35.57  (35.00-36.14) | 42.28  (40.92-43.78) | **0.001** |

^BPD: bronchopulmonary dysplasia. PDA: patent ductus arteriosus. ROP: retinopathy of prematurity. NEC: necrotizing enterocolitis. crUSS: cranial ultrasound. IVH: intraventricular haemorrage. PVL: periventricular leukomalacia. GA: gestational age. AKI: acute kidney injury. IQR: interquartile range.^
